# Postoperative locoregional recurrence pattern and treatment management of stage pT4 sigmoid colon cancer: a retrospective cohort study

**DOI:** 10.1186/s13014-022-02064-9

**Published:** 2022-05-13

**Authors:** Yaobin Lin, Shan Liu, Liang Hong, Lingdong Shao, Junxin Wu

**Affiliations:** 1 College of Clinical Medicine for Oncology, Fujian Medical University, 420 Fuma Rd, Jin’an District, Fuzhou, 350014 Fujian China; 2 Department of Hematology-Oncology, Fujian Children’s Hospital, Fuzhou, 350014 Fujian China; 3 Department of Radiation Oncology, Fujian Medical University Cancer Hospital, Fujian Cancer Hospital, Fuzhou, 350014 Fujian China

**Keywords:** Sigmoid colon cancer, Recurrence, Survival, Surgery, Radiotherapy

## Abstract

**Background:**

This study aimed to explore the pattern of locoregional recurrence after surgery in patients with non-metastatic stage pT4 sigmoid colon cancer and the role of adjuvant radiotherapy on survival.

**Methods:**

We retrospectively analyzed data from 208 patients who underwent surgery in our hospital. The patients were randomly divided into training and validation groups at a 1:1 ratio. Patients at high risk for locoregional recurrence were screened using Cox regression analysis. Based on the data of 2,886 patients in the Surveillance, Epidemiology, and End Results (SEER) database, the effect of adjuvant radiotherapy on overall survival (OS) and cancer-specific survival (CSS) was evaluated by Kaplan–Meier curves.

**Results:**

Of the 208 patients, 57 (27.4%) presented with locoregional recurrences (14 anastomotic and 43 abdominal or pelvic lymph node recurrences). Multivariate analysis showed that serum CEA, differentiation, lymph node dissection number, and N stage were independent predictors of locoregional recurrence-free survival (all *p* < 0.05). A risk-stratification model was constructed, and a total score of ≥ 6.5 points was considered the high-risk group for locoregional recurrence. Both the training and validation sets presented that the model had a good predictive ability (area under the curve = 0.828 and 0.724, respectively). Analysis of SEER data revealed that adjuvant radiotherapy significantly prolonged OS and CSS in the high-risk population (all *p* < 0.05, vs. no radiotherapy).

**Conclusions:**

Patients with a total risk score of 6.5 or more had a high likelihood of locoregional recurrence, and perhaps adjuvant radiotherapy could improve their survival.

**Supplementary Information:**

The online version contains supplementary material available at 10.1186/s13014-022-02064-9.

## Background

Colon cancer is the most common malignant disease of the gastrointestinal tract, with more than 100,000 newly diagnosed cases each year [1]. For locally advanced colon cancer, surgery alone or in combination with postoperative chemotherapy is the mainstay of treatment. However, the five-year overall survival (OS) rates of these patients were only 52–64% [2]. In recent decades, with the development of systemic therapies, the rate of distant metastasis in colon cancer has gradually decreased, and the rate of postoperative locoregional recurrence (defined as tumor relapses at the anastomotic site or within regional lymph nodes of the sigmoid colon) is between 10–40% [3–6]. Therefore, it is important to identify high-risk groups for locoregional recurrence and formulate treatment strategies.

The sigmoid colon, the last part of the colon, is especially prone to cancer and accounts for approximately 39.2% of all colon cancers [7]. For stage T4 sigmoid colon cancer, it is sometimes difficult to perform radical resection because of its anatomical characteristics [8–10]; On the other hand, it is often accompanied by invasion of adjacent organs or structures, requiring multiple organ resection. It has been reported that the proportion of R1/R2 resection (incomplete resection) is 15–35% [6, 11–13].

Radiotherapy (RT), another important local treatment method besides surgery, is not widely used in colon cancer [12, 14]. The sigmoid colon is much lower and easier to position on the RT target area delineation system than other parts of the colon, making adjuvant RT plausible for sigmoid colon cancer [15, 16]. Our previous study also found that adjuvant RT could prolong survival for stage pT4bN0-2M0 sigmoid colon cancer patients [17]. The National Comprehensive Cancer Network guideline of colon cancer indicates that postoperative RT can be considered for stage T4 with penetration into a fixed structure. However, it does not provide a more detailed description of the target population.

This study first analyzed the clinicopathological data of 208 patients with non-metastatic pathologic stage T4 (pT4) sigmoid colon cancer in Fujian Cancer Hospital and selected high-risk patients with locoregional recurrence after surgery using a risk-stratification model. Then, in 2,886 patients from the Surveillance, Epidemiology, and End Results (SEER) database, we preliminarily demonstrated that postoperative RT has a positive prognostic role in the population with high-risk of locoregional recurrence.

## Methods

### Patients

The medical records of patients with stage pT4 sigmoid colon cancer who received colectomy at our hospital from January 2010 to December 2016 were retrospectively analyzed. A total of 208 patients were included after excluding five patients who were lost to follow-up. Inclusion criteria were as follows: The patients (1) were diagnosed with sigmoid colon cancer by pathology, (2) received sigmoid colon resection, (3) had a postoperative stage was pT4N0-2M0, (4) received no preoperative neoadjuvant therapy, (5) received no other anti-tumor therapy except chemotherapy after surgery, and (6) provided complete follow-up information.

Clinicopathological data of stage pT4 sigmoid colon cancer patients diagnosed between 2004 and 2016 were screened from SEER database. Using SEER*Stat 8.3.8 (IMS, Inc. USA. https://seer.cancer.gov/seerstat/), cases were identified based on the third edition of the International Classification of Diseases for Oncology code, as described in a previous article [17]. The screening process is illustrated in Additional file 1. Finally, 2886 patients were included in the analysis. Both T stage and N stage were redefined based on the American Joint Committee on Cancer TNM staging classification for colon cancer 8th edition [18].

### Treatments and follow-up

All patients in our hospital underwent colectomy with en-bloc removal of the regional lymph nodes, of which 20 (9.6%) underwent combined organ resection due to tumor invasion. Dissection of lymph nodes from paracolic to the root of the inferior mesenteric artery, as well as suspicious enlarged lymph nodes observed on preoperative imaging. Complete mesocolic excision was performed, and surgical margins were negative in all patients. One hundred fifty-five patients (74.5%) received adjuvant chemotherapy. The three most common regimens were CAPEOX (capecitabine with oxaliplatin), FOLFOX (leucovorin, 5-fluorouracil, and oxaliplatin), and capecitabine single-agent chemotherapy. Follow-up data, including locoregional recurrence and survival status, were collected from medical records and telephone interviews. At the last follow-up, of the 208 patients, 98 were alive and 73 died. The remaining 37 patients could not be contacted because their contact information was changed. The latest follow-up information for these patients was recorded. Patients without postoperative follow-up records were excluded from the study. The median follow-up time was 61 months (interquartile range: 31–86 months).

All patients in the SEER database underwent surgery and divided into the surgery alone group (2,628 cases, 91.1%) and the surgery followed by adjuvant RT group (258 cases, 8.9%). Chemotherapy was not analyzed because the SEER database cannot distinguish which patients did not receive chemotherapy [19, 20]. All patients were actively followed-up. More detailed follow-up information was not available from the SEER database.

### Statistical analysis

SPSS Statistics (version 26.0, IBM Corp) and GraphPad Prism (version 5.0.1, GraphPad Software) were used for data analysis and plotting graphs. Statistical significance was set at P < 0.05.

The 208 patients screened from our hospital were equally and randomly assigned to training or validation groups using SPSS software (set seed = 2,000,000). Survival rates were calculated by Kaplan–Meier curves. Using Cox proportional risk regression, independent predictors associated with locoregional recurrence-free survival (LRRFS) were screened, and a model for predicting postoperative locoregional recurrence was constructed. The predictive power and optimal cutoff value of the model were assessed by receiver operating characteristic (ROC) curve and Youden index, respectively [21]. Data from the SEER database was analyzed to assess the effect of adjuvant RT on OS and cancer-specific survival (CSS) in groups at different risk of locoregional recurrence.

## Results

### Patient characteristics for our institution

The general characteristics of 208 patients are presented in Table 1. Patients aged 50–69 years accounted for 57.2% of the study population. The proportions of patients with elevated serum carcinoembryonic antigen (CEA) levels and ≥ 12 lymph node resections were 40.4% and 82.2%, respectively. The surgical margins were negative in all patients. Most patients had no vascular invasion (80.3%) or perineural invasion (81.7%). Finally, 74.5% of patients received postoperative adjuvant chemotherapy.

**Table 1:** Characteristics of patients with stage pT4 sigmoid colon cancer

Character	Training set (n = 104)		Validation set (n = 104)		Total (n = 208)	
	N	%	N	%	N	%
Age (year)						
< 50	31	29.8	17	16.3	48	23.1
50–69	52	50.0	67	64.4	119	57.2
≥ 70	21	20.2	20	19.2	41	19.7
Sex						
Male	66	63.5	62	59.6	128	61.5
Female	38	36.5	42	40.4	80	38.5
Serum CEA						
Normal	63	60.6	61	58.7	124	59.6
Elevated	41	39.4	43	41.3	84	40.4
Serum CA19-9						
Normal	73	70.2	78	75.0	151	72.6
Elevated	31	29.8	26	25.0	57	27.4
Type of surgery						
Colon resection	94	90.4	94	90.4	188	90.4
Multiorgan resection	10	9.6	10	9.6	20	9.6
Differentiation						
Well/moderate	88	84.6	85	81.7	173	83.2
Poor/undifferentiated	16	15.4	19	18.3	35	16.8
Tumor size (cm)						
< 5	49	47.1	52	50.0	101	48.6
≥ 5	55	52.9	52	50.0	107	51.4
Surgical margins						
Negative	104	100.0	104	100.0	208	100.0
Positive	0	0.0	0	0.0	0	0.0
Vascular invasion						
No	83	79.8	84	80.8	167	80.3
Yes	21	20.2	20	19.2	41	19.7
Perineural invasion						
No	82	78.8	88	84.6	170	81.7
Yes	22	21.2	16	15.4	38	18.3
Lymph node dissection number						
≥ 12	88	84.6	83	79.8	171	82.2
< 12	16	15.4	21	20.2	37	17.8
T stage						
T4a	88	84.6	95	91.3	183	88.0
T4b	16	15.4	9	8.7	25	12.0
N stage						
N0	35	33.7	56	53.8	91	43.8
N1	46	44.2	29	27.9	75	36.1
N2	23	22.1	19	18.3	42	20.2
Adjuvant chemotherapy						
Yes	83	79.8	72	69.2	155	74.5
No	21	20.2	32	30.8	53	25.5

*pT4* pathologic stage T4, *N* number, *CEA* carcinoembryonic antigen, *CA19-9* carbohydrate antigen 19-9

### Locoregional recurrence pattern of stage pT4 sigmoid colon cancer

**Fig. 1:**
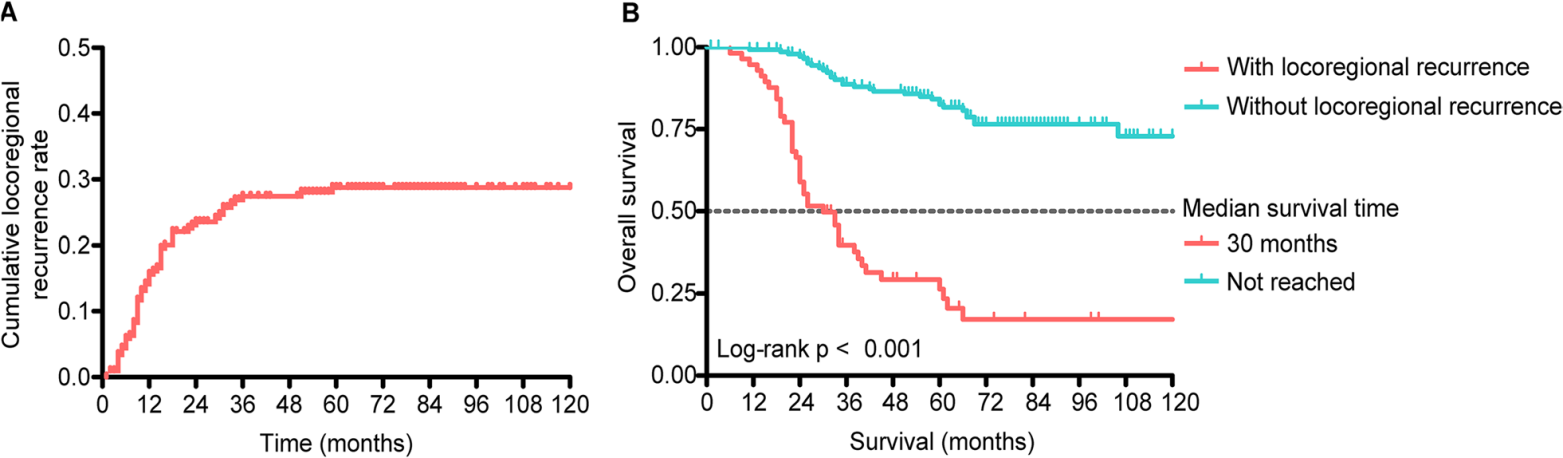
The cumulative locoregional recurrence rates and OS rates for patients with stage pT4 sigmoid colon cancer. **A** The cumulative locoregional recurrence rates after surgery. **B** OS rates for patients with or without locoregional recurrence. *OS* overall survival

Locoregional recurrence occurred in 57 of 208 patients (27.4%). Fourteen patients (6.7%) had an anastomotic recurrence, and 43 (20.7%) had abdominal or pelvic lymph node recurrence. The cumulative locoregional recurrence rates in the first, second and third years after surgery were 15.6, 23.6, and 26.5%, respectively (Fig. 1A). Locoregional recurrence mainly occurred within three years after surgery (96.5%, 55/57). The median locoregional recurrence time was 11 months, and the interquartile range was 8–18 months.

Once locoregional recurrence occurred, OS decreased significantly (*p* < 0.001, Fig. 1B). The 1-, 3-, and 5-year OS rates in the non-recurrence and recurrence groups were 99.3% vs. 94.7%, 88.7% vs. 39.8%, and 82.6% vs. 26.4%, respectively.

### Prognostic factors affecting LRRFS

Table 2 shows the results of the Cox regression analysis of independent predictors related to postoperative LRRFS of stage pT4 sigmoid colon cancer in the training set. Univariate analysis revealed that serum CEA, serum carbohydrate antigen 19–9 (CA19-9), differentiation, vascular invasion, lymph node dissection number, and N stage were associated with LRRFS (all *p* < 0.05). Multivariate analysis showed that elevated serum CEA (*p* < 0.001), poor/undifferentiated differentiation (*p* = 0.024), lymph node dissection number fewer than 12 (*p* = 0.029), and lymph node metastasis (*p* = 0.001) were related to poorer LRRFS. Serum CA19-9 levels and vascular invasion were not independent factors for LRRFS (all *p* > 0.05).

**Table 2:** Variables associated with LRRFS for stage pT4 sigmoid colon cancer in the training set

Variable	Univariable analysis		Multivariable analysis	
	Hazard ratio (95% CI)	P value	Hazard ratio (95% CI)	*P* value
Age (year)		0.077		
< 50	Reference	–		
50–69	0.744 (0.364–1.520)	0.417		
≥ 70	0.180 (0.040–0.797)	0.024		
Sex				
Male	Reference	–		
Female	0.781 (0.379–1.611)	0.504		
Serum CEA				
Normal	Reference	–	Reference	–
Elevated	4.765 (2.258–10.056)	< 0.001	4.844 (2.137–10.979)	< 0.001
Serum CA19-9				
Normal	Reference	–	Reference	–
Elevated	2.806 (1.414–5.567)	0.003	1.575 (0.762–3.256)	0.221
Type of surgery				
Colon resection	Reference	–		
Multiorgan resection	1.216(0.427–3.458)	0.714		
Differentiation				
Well/moderate	Reference	–	Reference	–
Poor/undifferentiated	3.256 (1.541–6.878)	0.002	2.630 (1.139–6.074)	0.024
Tumor size (cm)				
< 5	Reference	–		
≥ 5	1.382 (0.687–2.779)	0.365		
Vascular invasion				
No	Reference	–	Reference	–
Yes	2.311 (1.094–4.878)	0.028	1.326 (0.576–3.050)	0.507
Perineural invasion				
No	Reference	–		
Yes	1.220 (0.550–2.710)	0.624		
Lymph node dissection number				
≥ 12	Reference	–	Reference	–
< 12	3.332 (1.580–7.026)	0.002	2.789 (1.112–6.994)	0.029
T stage				
T4a	Reference	–		
T4b	0.964 (0.372–2.499)	0.940		
N stage		< 0.001		0.001
N0	Reference	–	Reference	–
N1	2.089 (0.735–5.936)	0.167	1.039 (0.320–3.378)	0.949
N2	8.138 (2.962–22.358)	< 0.001	4.741 (1.591–14.125)	0.005
Adjuvant chemotherapy				
Yes	Reference	–		
No	0.449 (0.158–1.280)	0.134		

*pT4* pathologic stage T4, *CI* confidence interval, *LRRFS* locoregional recurrence-free survival, *CEA* carcinoembryonic antigen, *CA19-9* carbohydrate antigen 19-9

### Establishment of the risk‑stratification model for LRRFS

Based on the β regression coefficient and Exp (B) value, which were generated from the Cox regression analysis, a risk scoring system was constructed (Table 3). The scoring system aimed to integrate factors identified from multivariate analysis: serum CEA level, differentiation, lymph node dissection number, and N stage. We assigned 5 points for elevated serum CEA level, 3 points for poorly differentiated or undifferentiated cells, 3 points for < 12 lymph nodes dissected, and 5 points for the N2 stage. Zero points were assigned to normal serum CEA level, well/moderate differentiation status, removal of fewer than 12 lymph nodes, and the N0 and N1 stages.

**Table 3:** Risk scoring system

Risk variable	B value	Exp (B)	Risk coefficient	Risk score
Serum CEA				
Normal	0.000	1.000	1.000	0
Elevated	1.578	4.844	4.844	5
Differentiation				
Well/moderate	0.000	1.000	1.000	0
Poor/undifferentiated	0.967	2.630	2.630	3
Lymph node dissection number				
≥ 12	0.000	1.000	1.000	0
< 12	1.026	2.789	2.789	3
N stage				
N0	0.000	1.000	1.000	0
N1	0.039	1.039	1.039	0
N2	1.556	4.741	4.741	5

*CEA* carcinoembryonic antigen

**Fig. 2:**
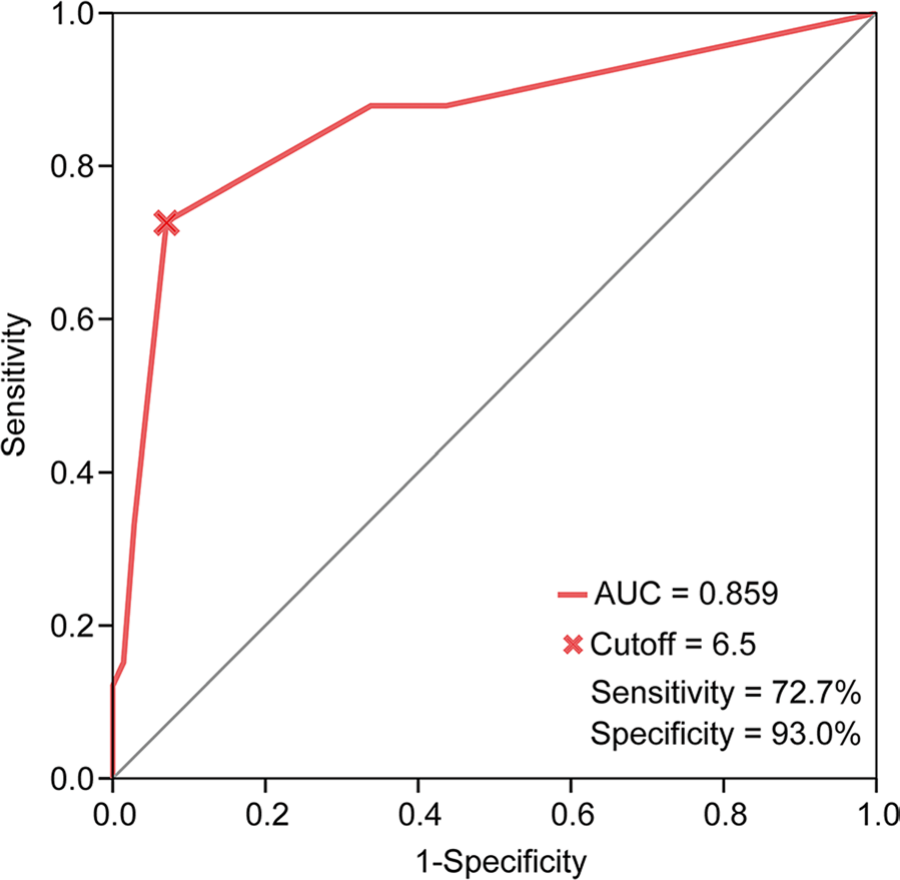
ROC curve of risk scores. The optimal cutoff value was assessed for the event of locoregional recurrence. *ROC* receiver operating characteristic; *AUC* area under the curve

Each patient in the training set was scored according to the risk scoring system, and the total score was calculated. Figure 2 showed the area under the curve (AUC) was 0.859 with an optimal cutoff value of 6.5 (sensitivity = 72.7%, specificity = 93.0%).

### Valuation of the risk‑stratification model

**Fig. 3:**
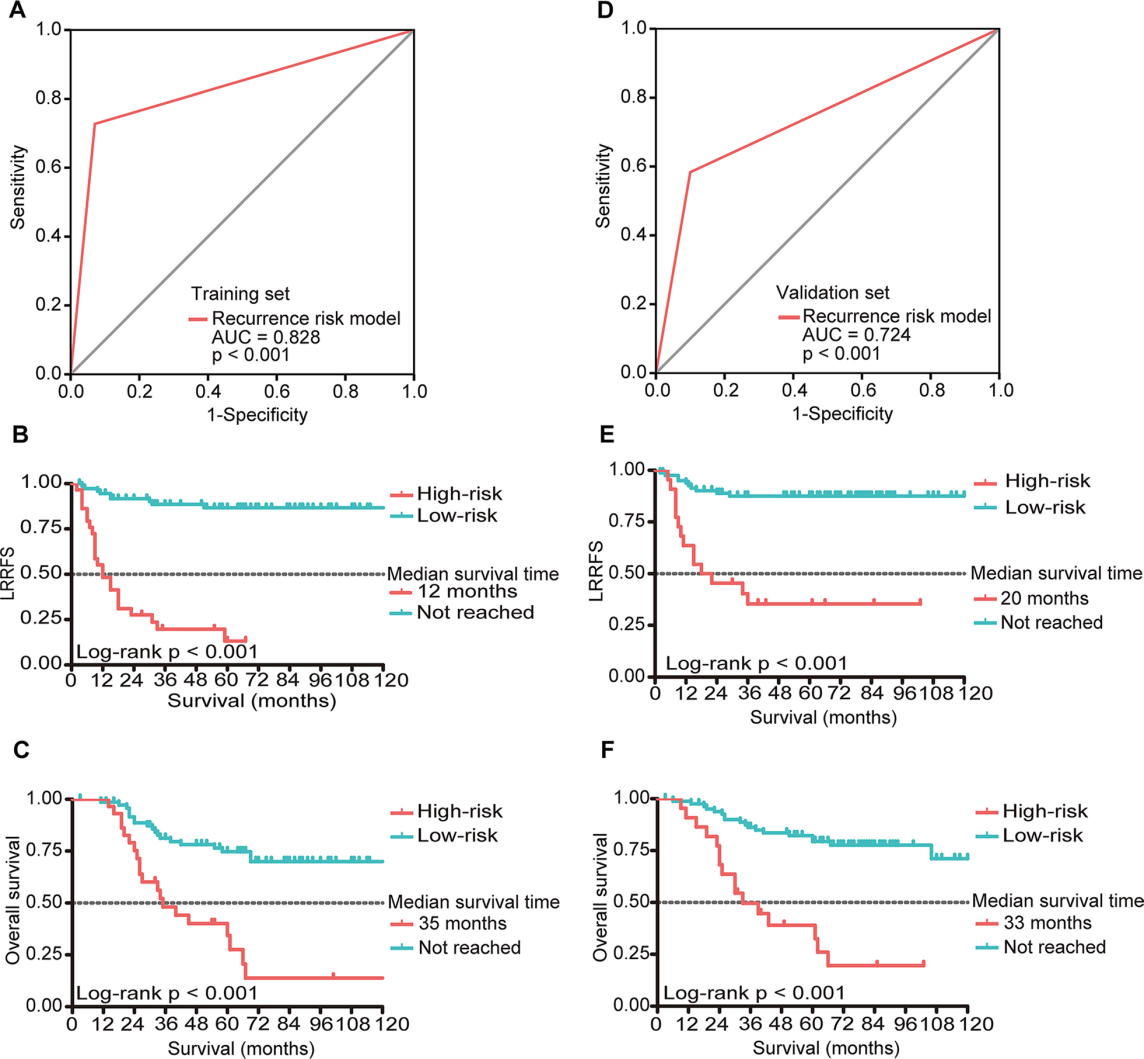
Valuation of the risk-stratifications model in training and validation set. **A** ROC curve of risk stratifications in the training set. **B** LRRFS rates of different risk stratifications in the training set. **C** OS rates of different risk stratifications in the training set. **D** ROC curve of risk stratifications in the validation set. **E** LRRFS rates of different risk stratifications in the validation set. **F** OS rates of different risk stratifications in the validation set. *ROC* receiver operating characteristic; *AUC* area under the curve; *LRRFS* locoregional recurrence-free survival; *OS* overall survival

For training set, 75 (72.1%) patients were classified into the low locoregional recurrence risk group (total score < 6.5), and 29 (27.9%) were classified into the high risk group (total score ≥ 6.5). The AUC of this risk‑stratification model is 0.828 (*p* < 0.001, Fig. 3A). Figure 3B reveals that the high-risk group had worse LRRFS than the low-risk group. Their 1-, 3-, and 5-year LRRFS rates were 48.3% vs. 94.5%, 19.7% vs. 88.6%, and 13.1% vs. 86.7% (*p* < 0.001), respectively. In addition, OS was also worse in the high-risk group (*p* < 0.001, Fig. 3C).

The patients in the validation set were scored and grouped according to the model. It also showed a good predictive power (AUC = 0.724, *p* < 0.001, Fig. 3D). Similar to the training set, the LRRFS and OS were worse in the high-risk group (all* p* < 0.001, vs. low-risk group, Fig. 3E, F).

### Patient characteristics for SEER database

A total of 2,886 patients with non-metastatic stage pT4 sigmoid colon cancer were analyzed from the SEER database (Table 4). Most patients from the SEER database were also aged 50–69 years (47.7%). Patients with elevated serum CEA levels, poor differentiation/undifferentiated tumor status, < 12 lymph nodes removed, and lymph node metastases were 52.2%, 21.8%, 19.3%, and 56.9%, respectively.

**Table 4:** Characteristics of patients with stage pT4 sigmoid colon cancer from SEER database

Variable	With adjuvant radiation (n = 258)		Without adjuvant radiation (n = 2628)		Total (n = 2886)	
	N	%	N	%	N	%
Insurance						
Insured	185	71.7	2003	76.2	2188	75.8
Uninsured	18	7.0	106	4.0	124	4.3
Unknown	55	21.3	519	19.7	574	19.9
Age (year)						
<50	57	22.1	468	17.8	525	18.2
50–69	144	55.8	1233	46.9	1377	47.7
≥ 70	57	22.1	927	35.3	984	34.1
Sex						
Male	129	50.0	1360	51.8	1489	51.6
Female	129	50.0	1268	48.2	1397	48.4
Race						
White	196	76.0	1984	75.5	2180	75.5
Black	22	8.5	289	11.0	311	10.8
Others	39	15.1	343	13.1	382	13.2
Unknown	1	0.4	12	0.5	13	0.5
Marital status						
Married	140	54.3	1348	51.3	1488	51.6
Others	107	41.5	1175	44.7	1282	44.4
Unknown	11	4.3	105	4.0	116	4.0
Differentiation						
Well/moderate	211	81.8	2046	77.9	2257	78.2
Poor/undifferentiated	47	18.2	582	22.1	629	21.8
Tumor size (cm)						
< 5	66	25.6	1096	41.7	1162	40.3
≥ 5	183	70.9	1462	55.6	1645	57.0
Unknown	9	3.5	70	2.7	79	2.7
Serum CEA						
Normal	119	46.1	1261	48.0	1380	47.8
Elevated	139	53.9	1367	52.0	1506	52.2
Perineural Invasion						
Yes	33	12.8	379	14.4	412	14.3
No	108	41.9	1154	43.9	1262	43.7
Unknown	117	45.3	1095	41.7	1212	42.0
Lymph node dissection number						
≥ 12	212	82.2	2116	80.5	2328	80.7
< 12	46	17.8	512	19.5	558	19.3
T stage						
T4a	83	32.2	1653	62.9	1736	60.2
T4b	175	67.8	975	37.1	1150	39.8
N stage						
N0	148	57.4	1095	41.7	1243	43.1
N1	66	25.6	850	32.3	916	31.7
N2	44	17.1	683	26.0	727	25.2

N = number; CEA = carcinoembryonic antigen; SEER = the Surveillance, Epidemiology, and End Results

### Effect of adjuvant radiotherapy on survival in different locoregional recurrence risk groups

**Fig. 4:**
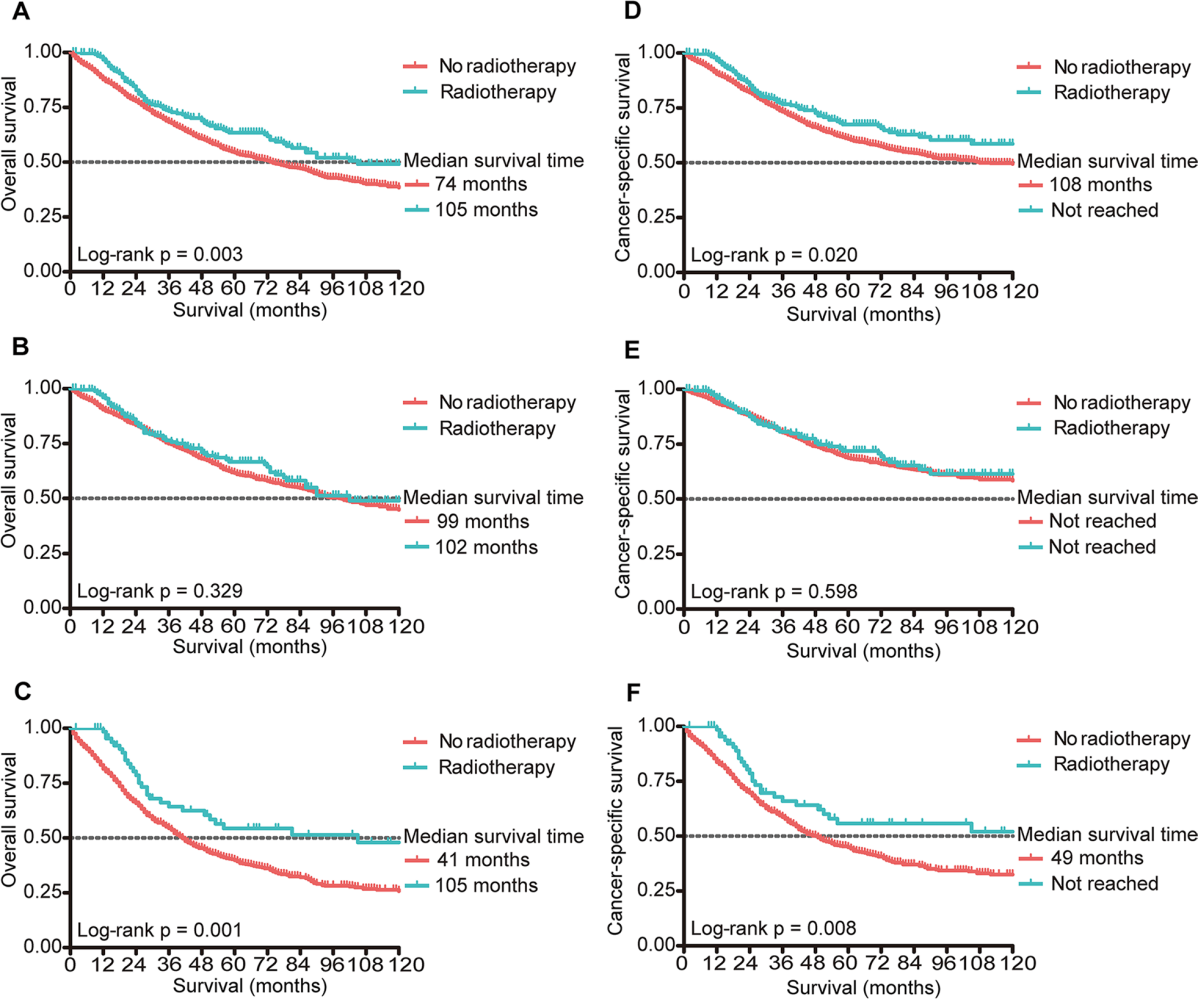
The effect of adjuvant radiotherapy on OS and CSS. OS rates of all patients (**A**), low-risk group (**B**), and high-risk group (**C**). CSS rates of all patients (**D**), low-risk group (**E**), and high-risk group (**F**). *OS* overall survival; *CSS* cancer-specific survival

Based on the model, 1973 (68.4%) patients were assigned to the low-risk group and 913 (31.6%) patients to the high-risk group. The effect of adjuvant RT on OS are presented in Fig. 4A–C. Adjuvant RT significantly prolonged OS in the entire population (Fig. 4A, *p* = 0.003). For high-risk patients, adjuvant RT improved the OS, and the median OS was prolonged by 64 months (Fig. 4C, *p* = 0.001). While for patients with low risk of locoregional recurrence, the effect of adjuvant RT on OS was not significant (Fig. 4B, *p* = 0.329).

The effect of adjuvant RT on CSS are presented in Fig. 4D–F. Similar to OS, adjuvant RT extended CSS in the entire population (Fig. 4D, *p* = 0.020) and high-risk group (Fig. 4F, *p* = 0.008). For patients with low locoregional recurrence risk, treatment with or without adjuvant RT had no significant impact on CSS (Fig. 4E, *p* = 0.598).

## Discussion

In this study, a risk-stratification model was established to screen the high-risk population of postoperative locoregional recurrence. The model integrated four parameters: serum CEA level, differentiation status, number of lymph nodes dissected, and N stage. Compared with the low-risk group, high-risk patients (total score ≥ 6.5) were more likely to have a locoregional recurrence and worser LRRFS and OS (all *p* < 0.001). In addition, this study confirmed that adjuvant RT could improve OS and CSS in patients with high locoregional recurrence risk (all *p* < 0.05, vs. no RT). The model we developed may have implications for the postoperative management of some patients, such as providing local interventions and strengthening follow-up monitoring.

Locoregional recurrence or distant metastasis of colon cancer mainly occurs within 3 years after surgery [22], and Park et al. [23] reported that patients who relapsed within 3 years after surgery accounted for 95% of the total population with recurrence. Similar results were also found in this study; the cumulative locoregional recurrence rate increased rapidly in the first 2 years, and patients who relapsed within 3 years accounted for 96.5% of the recurrence population. Even if the surgical margins of the patients were all negative, 6.7% of patients had an anastomotic recurrence. However, the factors affecting locoregional recurrence have not been determined, and the results of different studies have been slightly different. Wang et al. [24] focused on the tumor site, T stage, and serum CEA level. Vergara-Fernandez et al. [25] suggested that the number of lymph nodes removed and nerve invasion are important elements affecting the locoregional recurrence of colorectal cancer. Analysis of sex, age, and family history of cancer showed no correlation with postoperative recurrence or metastasis of colon cancer [26]. It demonstrated that colon cancer locoregional recurrence may be affected by many complex factors. This study also confirmed that serum CEA level (*p* < 0.001) and the lymph node dissection number (*p* = 0.029) were independent factors affecting locoregional recurrence. For differentiation and N stage, they were also the independent predictors (*p* = 0.024 and *p* = 0.001, respectively). Adjuvant chemotherapy was ineffective at local tumor control (*p* = 0.134), requiring additional postoperative treatments such as RT.

Serum CEA is a routinely-used marker for diagnosing and monitoring colorectal cancer [27, 28]. However, different researchers hold different views on the role of serum CEA for prognosis [19, 24, 29]. In our current research, elevated serum CEA was found to be an independent predictor of locoregional recurrence (*p* < 0.001), with a score of 5 in the risk stratification model. Therefore, regular monitoring of changes in serum CEA levels after surgery is still recommended.

Another key factor affecting the survival of colon cancer patients is lymph node metastasis, which is also a determinant of postoperative treatment [2, 30, 31]. As confirmed in this study, the N stage was associated with postoperative locoregional recurrence (*p* = 0.001), and the risk in stage N2 patients was 4.741 times than that in stage N0 patients (95% confidence interval [CI]: 1.591–14.125, *p* = 0.005). However, the risk of locoregional recurrence in stage N1 patients was similar to the stage N0 patients (*p* = 0.949). Therefore, patients with stage N2 disease should be vigilant about locoregional recurrence after surgery so that it can be treated as soon as possible. In addition to the N stage, we found that the lymph nodes dissected number was related to locoregional recurrence. The risk of locoregional recurrence was 2.789-fold higher in patients with fewer than 12 lymph node dissection (95% CI: 1.112–6.994, *p* = 0.029). Surgeons should try their best to remove 12 or more lymph nodes, as required by clinical guidelines, to achieve a more accurate N stage and reduce the risk of postoperative locoregional recurrence.

Perineural invasion is a feature of aggressiveness for colon cancer [24, 32]. It is reported that invasion of peripheral nerves means worse five-year survival [33]. This conclusion has also been confirmed by other studies [25, 34–36]. However, recent studies have reached contradictory conclusions, finding that perineural infiltration is unrelated to locoregional recurrence or poor prognosis of colon cancer [4, 24]. In our study, on the premise that surgical margins were negative, it was also found that perineural invasion was not affecting the locoregional recurrence (*p* = 0.624).

RT is one of the most important local interventions; however, for colon cancer, postoperative RT is rarely performed. Preliminary studies conducted in the 1990s revealed that adjuvant RT could improve local control and disease-free survival (DFS) of colon cancer [13, 37, 38]. Since 2000, interest in RT has diminished [39]. This may be related to the lack of prospective clinical studies that clearly support the role of RT in colon cancer. The only phase III randomized controlled trial had shown similar survival rates for postoperative chemoradiotherapy and chemotherapy in colon cancer (5-year OS: 58% vs. 62%, *p* > 0.05; 5-year DFS: 51% vs. 51%, *p* > 0.05) [14]. At the same time, the trial had several limitations, such as the inclusion of T3 patients (18.7%, 35/187) and insufficient enrollment (the original plan was to enroll 700 cases, and enrollment was stopped after 222 cases). Another shortcoming was that only 48% of patients underwent preoperative imaging, and 19% of patients had clips placed during the operation, making the implementation of postoperative RT more difficult.

As targeted therapy and immunotherapy are more widely adopted, the pattern of treatment failure has changed [40], and the distant failure rate has decreased. With further exploration of the efficacy and safety of chemoradiotherapy for colon cancer, the role of radiation is being reconsidered [41–43]. A single-center retrospective study reported in 2016 that adjuvant RT can enhance the local control rate and prolong DFS, particularly in patients with stage T4b or residual tumors [6]. These findings have also been confirmed by other studies [12, 44]. Previously, we also found that some sigmoid colon cancer patients may benefit from postoperative RT [17], and this study once again demonstrated the positive effect of adjuvant RT on survival from the perspective of locoregional recurrence.

Adjuvant RT has several advantages for sigmoid colon cancer compared to other parts of the colon. First, the sigmoid colon extends from the left iliac crest to the third sacral plane and continues with the rectum, which can be readily positioned and targeted for precise local irradiation [15, 16]. In other parts of the colon, it is difficult to delineate the postoperative target area under the condition of organ movement without the placement of silver clips [4, 42, 45]. Second, the types of organs at risk for sigmoid colon cancer are relatively fixed, which facilitates quality control of RT. Third, RT in the surgical area of sigmoid colon cancer has a smaller irradiation volume and a lower cumulative radiation dose to the small intestine, which may reduce the occurrence of radiation enteritis. In addition, adjuvant RT for colon cancer was previously performed using two-dimensional total abdominal irradiation [46–48], while the application of three-dimensional conformal RT and intensity-modulated RT techniques has reduced radiation toxicity in normal tissues such as the small bowel [15, 16]. The development of motion management technology also provides technical support for RT in colon cancer [45].

This study had some limitations. First, the cases used to construct the risk stratification model were all negative for the surgical margins. Therefore, this model is not suitable for predicting the risk of locoregional recurrence in patients with positive resection margins; however, therapeutic intervention should be actively administered. Second, the selection method of variables included in the multivariate analysis has limitations. Factors with *p* values greater than 0.05 in the univariate analysis were not included in the multivariate analysis, but these factors may also be associated with locoregional recurrence. Third, the number of patients with colon cancer who received postoperative RT in our institution was not enough for statistical analysis, and there was no locoregional recurrence-related information in the SEER database [49]. Therefore, this study used the data from our center to analyze postoperative locoregional recurrence and then analyzed the effect of adjuvant RT on OS and CSS using the SEER database. It is necessary to conduct new studies to evaluate the value of postoperative RT from the perspective of LRRFS. In addition, the SEER database does not record details on RT, such as the range of irradiation target, prescribed dose, and radiation-related toxicity [20, 50]. Therefore, the details of the RT plan need to be explored further. Finally, it is difficult to avoid selection bias in retrospective analyses, which must be verified and further developed in prospective studies.

## Conclusions

In this study, a risk-stratification model was established for predicting postoperative locoregional recurrence in pT4 sigmoid colon cancer. This model has the advantages of few indices, easy accessibility, and simple operation. In this model, patients with a total score of 6.5 or more have a high probability of locoregional recurrence and poor survival. Local interventions, such as postoperative RT, should be supplemented for the high-risk population.

## Supplementary Information

**Additional file 1.** Flow chart of the search protocol and study design for SEER database. SEER, the Surveillance, Epidemiology, and End Results; CEA, carcinoembryonic antigen; OS, overall survival; CSS, cancer-specific survival

## Data Availability

The dataset analyzed in this study from SEER database can be obtained from: https://seer.cancer.gov/data/. Other data used and/or analyzed during the current study are available from the corresponding author on reasonable request.

## Abbreviations

OS: Overall survival
CSS: Cancer-specific survival
LRRFS: Locoregional recurrence-free survival
DFS: Disease-free survival
pT4: Pathologic stage T4
RT: Radiotherapy
CEA: Carcinoembryonic antigen
CA19-9: Carbohydrate antigen 19-9
SEER: The surveillance, epidemiology, and end results
ROC: Receiver operating characteristic
AUC: Area under the curve
HR: Hazard ratio
CI: Confidence interval
