# Supplementary material for: Postoperative locoregional recurrence pattern and treatment management of stage pT4 sigmoid colon cancer: a retrospective cohort study
Source: Radiat Oncol. 2022 May 13;17:95. doi: 10.1186/s13014-022-02064-9 (PMC9107167; doi:10.1186/s13014-022-02064-9)
Supplement: Supplementary file 1 — Additional file 1. Flow chart of the search protocol and study design for SEER database. SEER, the Surveillance, Epidemiology, and End Results; CEA, carcinoembryonic antigen; OS, overall survival; CSS, cancer-specific survival [file 13014_2022_2064_MOESM1_ESM.docx]

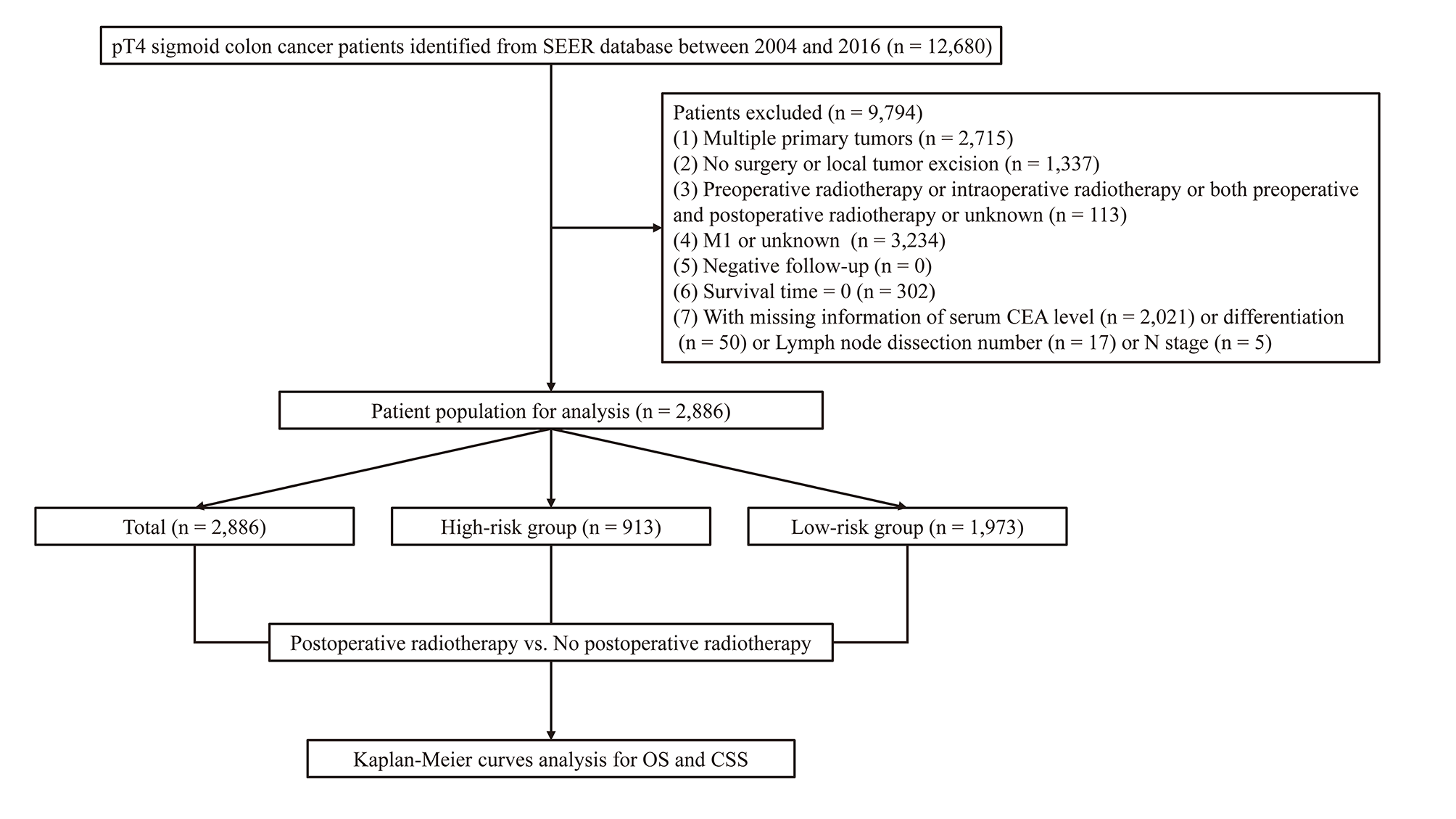


**Additional file 1.** Flow chart of the search protocol and study design for SEER database. SEER, the Surveillance, Epidemiology, and End Results; CEA, carcinoembryonic antigen; OS, overall survival; CSS, cancer-specific survival.
